# Intracranial artery calcification as an independent predictor of ischemic stroke: a systematic review and a meta-analysis

**DOI:** 10.1186/s12883-023-03069-x

**Published:** 2023-01-16

**Authors:** Xuelong Li, Heng Du, Jia Li, Xiangyan Chen

**Affiliations:** 1 Department of Health Technology and Informatics, The Hong Kong Polytechnic University, Kowloon, Hong Kong; 2 Department of Neurology, Wuhan No.1 Hospital, Tongji Medical College, Huazhong University of Science and Technology, Wuhan, Hubei China

**Keywords:** Intracranial artery calcification, Ischemic stroke, meta-analysis, Stroke occurrence; stroke recurrence; mortality

## Abstract

**Background and purpose:**

The association between intracranial artery calcification (IAC) and the risk of ischemic stroke occurrence or poor prognosis had not yet been fully understood. In this study, we conducted a meta-analysis of existing studies aimed to assess whether IAC can be used to predict future ischemic stroke and post-stroke mortality.

**Methods:**

Medline, Cochrane, Web of Science and Google Scholar databases were searched up to June 30, 2022. Studies were included if they reported risk ratio (RR) or odds ratios (OR) and corresponding 95% confidence intervals (CI) of stroke concerning the presence of IAC. Random or fixed effects model meta-analyses were performed. Meta-analysis was conducted by using Stata version 16.0.

**Results:**

Twelve studies involving 9346 participants were included. Compared with those without IAC, patients with IAC had a higher risk of stroke occurrence (adjusted OR 1.62, 95% CI 1.18–2.23, *P* = 0.001) and stroke recurrence (adjusted OR 1.77, 95% CI 1.25–2.51, *P* = 0.003). However, we did not find a significant correlation between IAC and post-stroke mortality (pooled OR 1.12, 95% CI 0.80–1.56, *P* = 0.504).

**Conclusions:**

Our meta-analysis demonstrated that the presence of IAC was identified as an independent risk factor for ischemic stroke occurrence and recurrence but is not a predictor of post-stroke mortality.

## Introduction

Stroke has remained in the top three causes of death and a major cause of disability globally, and its absolute number of cases increased substantially from 1990 to 2019 [1]. According to a population-based screening project in China, the prevalence of stroke in China and most provinces has continued to increase in the past 7 years (2013 − 2019) [2]. It has been shown that major arteries’ intracranial atherosclerotic stenosis (ICAS) represents a common cause of ischemic stroke worldwide. Intracranial arterial calcification (IAC), which is considered an active process of atherosclerosis, was first observed by ex vivo radiography and microscopic pathology in the early 1960s. According to our previous clinical studies, IAC was a highly prevalent finding on brain computed tomography (CT) scans among the general populations and patients with stroke or transient ischemic attack.

IAC is known to be an essential risk factor for cerebral infarction and an essential marker of ICAS [3–5]. Several recent studies have shown a significant association between IAC and first-ever ischemic stroke risk. Moreover, the value of IAC as a predictor of recurrent stroke risk was also demonstrated in these studies. Assessing IAC by using quantitative Agatston score, our recent study found that IAC was a strong risk factor for recurrent stroke and post-stroke mortality [6]. However, it is important to note that the association between calcification and stroke has not gone undisputed and these findings have never been pooled before. To provide clarity, we aim to inform the relationships between IAC and future ischemic stroke or mortality in the form of a meta-analysis of available studies.

## Methods

This study was preformed according to the Preferred Reporting Items for Systematic Reviews and Meta-Analyses (PRISMA) statement. It was registered in PROSPERO international prospective register of systematic reviews **(CRD42021281357)**.

### Data sources and search strategy

We searched Medline, Cochrane, Web of Science, Google Scholar databases from inception to June 30, 2022. Prospective or retrospective cohort and case-control studies were included. Relevant keywords, phrases and medical subject headings (MeSH) terms were used. The following search keywords in different combination were used: stroke, intracranial artery, calcification or calcified or calcium. Furthermore, we hand-searched reference lists of included studies to find additional studies. Only English language studies and accompanied by full-length peer-reviewed papers were included. Two reviewers independently screened and performed parallel assessments of the manuscripts. The following search strategy was used for Web of Science and modified to suit other databases:

#1 stroke.

#2 intracranial artery.

#3 calcification OR calcified OR calcium.

#4 #1 AND #2 AND #3.

We also used the “cited by” function of Google Scholar to minimize the risk of missing data.

### Study selection

Studies screened and examined by titles and abstracts after removing over-lapping and duplicate articles. Only studies that met the following criteria were included in this meta-analysis: limited to human subjects; report risk ratio (RR) or odds ratios (OR) and their corresponding 95% confidence intervals (CI) of stroke relating to IAC, and at least one of the following outcomes should be reported: occurrence, recurrence or mortality. Letters, comments, editorials, case reports, proceedings, and personal communications were excluded.

### Data extraction

Data extraction was independently performed by two reviewers using a standard form. The data extracted included the following information: first author, study design, publication year, country of origin, sample population demographic characteristics, gender, sample size, endpoint, and covariates adjustment in each study.

### Quality assessment

The quality assessment for observational studies included in this meta-analysis were assessed using the Newcastle-Ottawa Scale (NOS) [7]. This scale comprised of three domains (selection, comparability, and outcome) with a maximum score of 9 points. Thus, the risk of bias categorized into three groups: high (0-3), moderate (4-6), and low (7-9).

### Statistical analysis

Meta-analysis was performed using either fixed-effects models (I^2^ < 50.0%) or, in the presence of heterogeneity (I^2^ > 50.0%), random-effects models. Heterogeneity across studies was assessed by using both the Q test and the I^2^ statistic (ranging from 0% for perfect homogeneity to 100% for extreme heterogeneity). An I^2^ value > 50% indicates significant heterogeneity. We also performed a subgroup analysis to further assess the association between IAC and the risk of stroke events according to clinical characteristics, sample size and study design. Pooled effects were calculated, and a two-sided *P* value < 0.05 was generally considered to indicate statistical significance. Publication bias was not performed as enough studies are necessary for this type of analysis. The data analysis was done using Stata 16.0 software.

## Results

### Data sources and searches

**Fig. 1:**
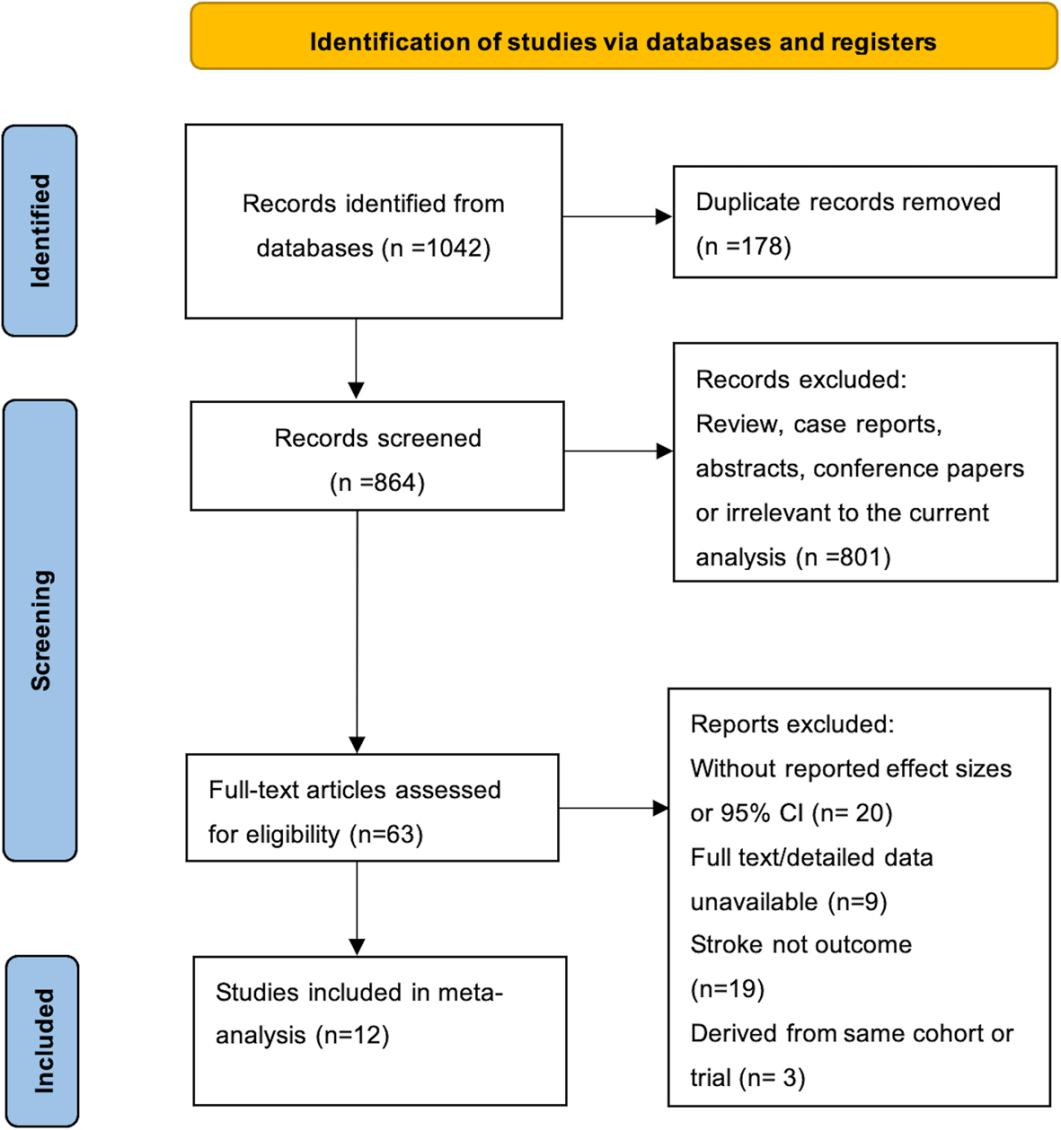
Flow chart for study screening and selection

Our initial electronic and hand search of all the databases identified 1042 records. After initial screening of the titles and abstracts, we reviewed the full text of the remaining 63 articles, and rejected 29 citations that did not report the effect sizes or full detailed data, 19 were excluded because the clinical outcome was not stroke and 3 further studies because they used the same data source. Overall, a total of 12 studies involving 9346 participants were eligible for quantitative synthesis (meta-analysis) [6, 8–18]. The flow chart for study inclusion is shown in Fig. 1.

### Study characteristics of included studies

The main characteristics of the included studies were presented in Table 1. Publication dates ranged from 2003 to 2020; the sample sizes of included studies ranged from 99 to 2323 participants; the average age of subjects was 67.3 years (range 51.0–73.6 years). Among these 12 studies, eight reported the risk of stroke occurrence, four reported the association between IAC and post-stroke mortality, only 3 reported the risk of stroke recurrence, two were from the United States, six were from Europe, and four were from Asia. The quality assessment of included study is presented in Table 1. The risk of bias for all 12 studies included in the meta-analysis was moderate or above with high quality.

**Table 1:** Characteristics of included studies

Trial	Region	Cases	Trial design	Age (years)	Male (%)	Outcome variables	aOR (95% CI)	Risk of bias
Douglas 2003	USA	322	Cohort study	73	53	OC	0.96 (0.1-8.97)	Moderate
Chen 2007	CHINA	357	Case-control study	65.9	54.1	OC	3.17 (1.25-8.04)	Moderate
Bugnicourt 2009	France	511	Case-control study	65.7	56.8	OC	1.89 (1.13-3.14)	Moderate
POWER 2011	UK	529	Cohort study	59	61	OC & MO	OC: 1.48 (1-2.23) MO: 2.17 (1.22–3.87)	Low
Koton 2012	Israel	1049	Cohort study	70	59	MO	Mild: 1.6 (0.6–4.3) Severe: 1.0 (0.4–3.0)	Moderate
Lee 2014	Korea	1017	Cohort study	67.7	56.7	RE & MO	Mild: RE: 1.49 (.83-2.67) MO: 0.51 (.19-1.37) Severe: RE: 2.00 (1.07-3.71) MO: 0.54 (.19-1.53)	Moderate
Bos2014	Europe	2323	Cohort study	69.5	47.8	OC	1.39 (0.98-1.99)	Moderate
Kao 2015	Netherlands	1872	Case-control study	NA	53.7	OC	1.02 (0.98, 1.07)	Low
Quiney 2017	USA	99	Case-control study	55	58.6	OC	2.2(1.2-3.9)	Moderate
Zhang 2019	China	125	Case-control study	NA	NA	OC	1.98 (1.45 ~ 2.69)	Low
Magdiˇ 2020	Slovenia	448	Case-control study	76	47.3	RE & MO	RE: 3.13 (1.35-7.20) MO: 1.22 (0.93-1.56)	Moderate
Wu 2020	China	694	Cohort study	71.6	50.3	RE & MO	RE: 1.23 (0.57, 2.66) MO: 3.17 (0.42-23.79)	Low

Abbreviations: *USA* United States of America, *NA* not available, *OC* occurrence, *RE* recurrence, *MO* mortality, *aOR* adjusted odds ratio, *CI* confidence intervals

### Association between IAC and ischemic stroke incidence

**Fig. 2:**
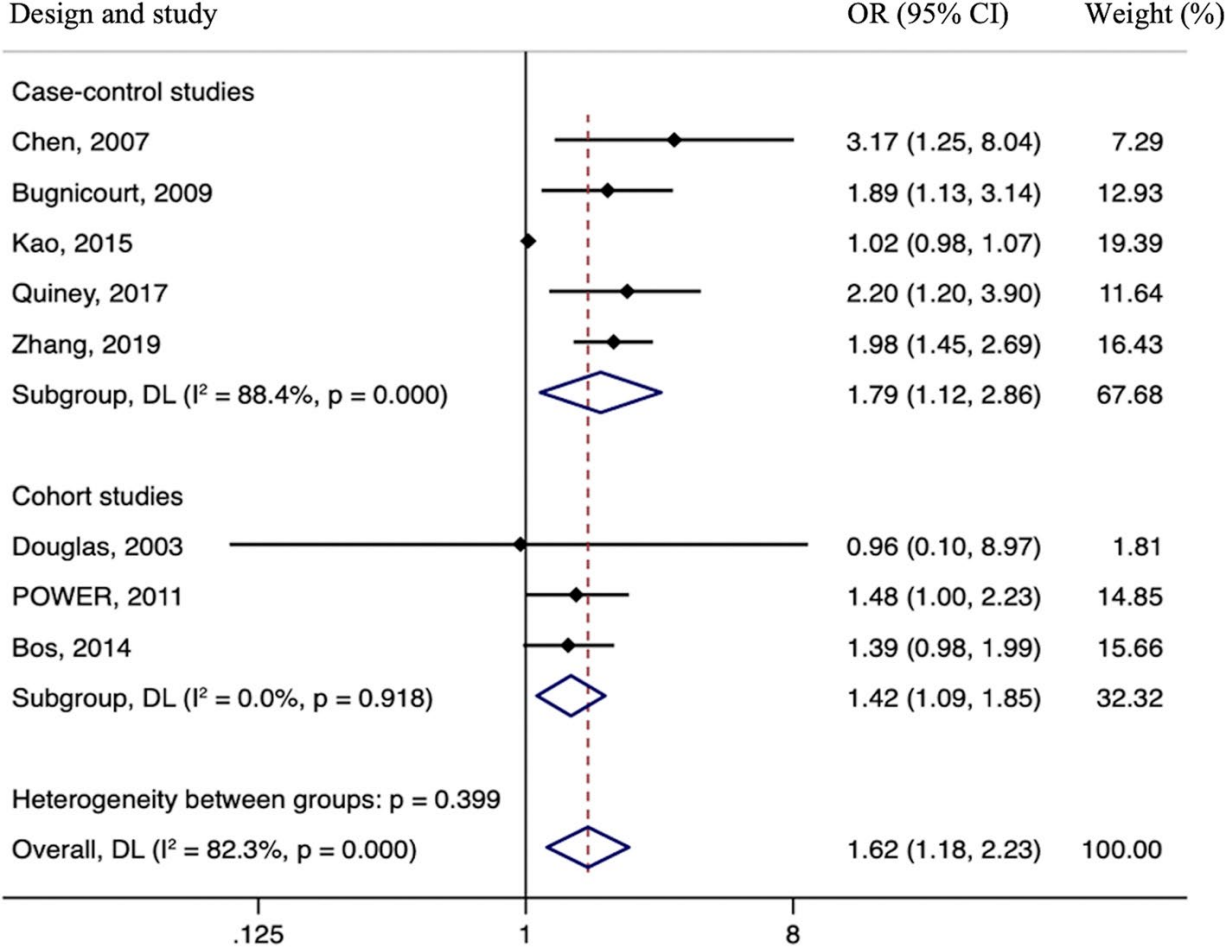
Meta-analyses of hazard ratios for the association between intracranial arterial calcification and stroke incidence

Compared with those without IAC, among subjects with IAC had an elevated risk of ischemic stroke (OR 1.62; 95% CI 1.18–2.23, *p* = 0.003; Fig. 2). A random-effects model was used to assess the pooled outcome due to the included studies’ extreme heterogeneity (I^2^ = 82.3%, *P* <  0.001). Considering the relatively high heterogeneity, a sensitivity analysis following the leave-one-out approach was performed to show that Kao et al. study had the most significant influence on the heterogeneity, and the pooled RR without this study was 1.75 (95% CI, 1.47–2.08) with the I^2^ value reduced to 0%. No associations between the modifiers age, study design, sample size, country, study quality and IAC were identified in meta-regression analyses (*p* = 0.46, *p* = 0.32, *p* = 0.25, *p* = 0.13, and *p* = 0.20, respectively).

### Association between IAC and ischemic stroke recurrence

**Fig. 3:**
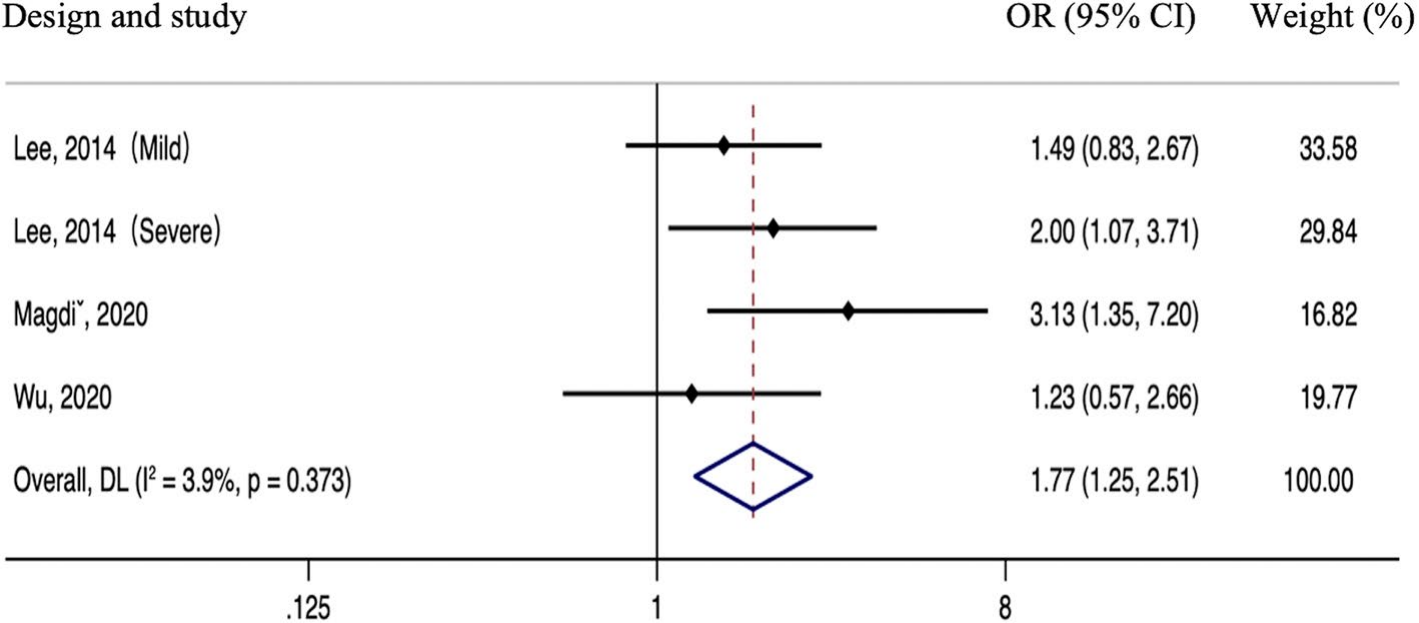
Meta-analyses of hazard ratios for the association between intracranial arterial calcification and stroke recurrence

Results from three studies demonstrated that the presence of IAC related to a higher risk of stroke recurrence (OR 1.77; 95% CI, 1.25–2.51, *p* = 0.001; Fig. 3) without significant heterogeneity (*P* = 0.373, I^2^ = 3.9%).

### Association between IAC and post-stroke mortality

**Fig. 4:**
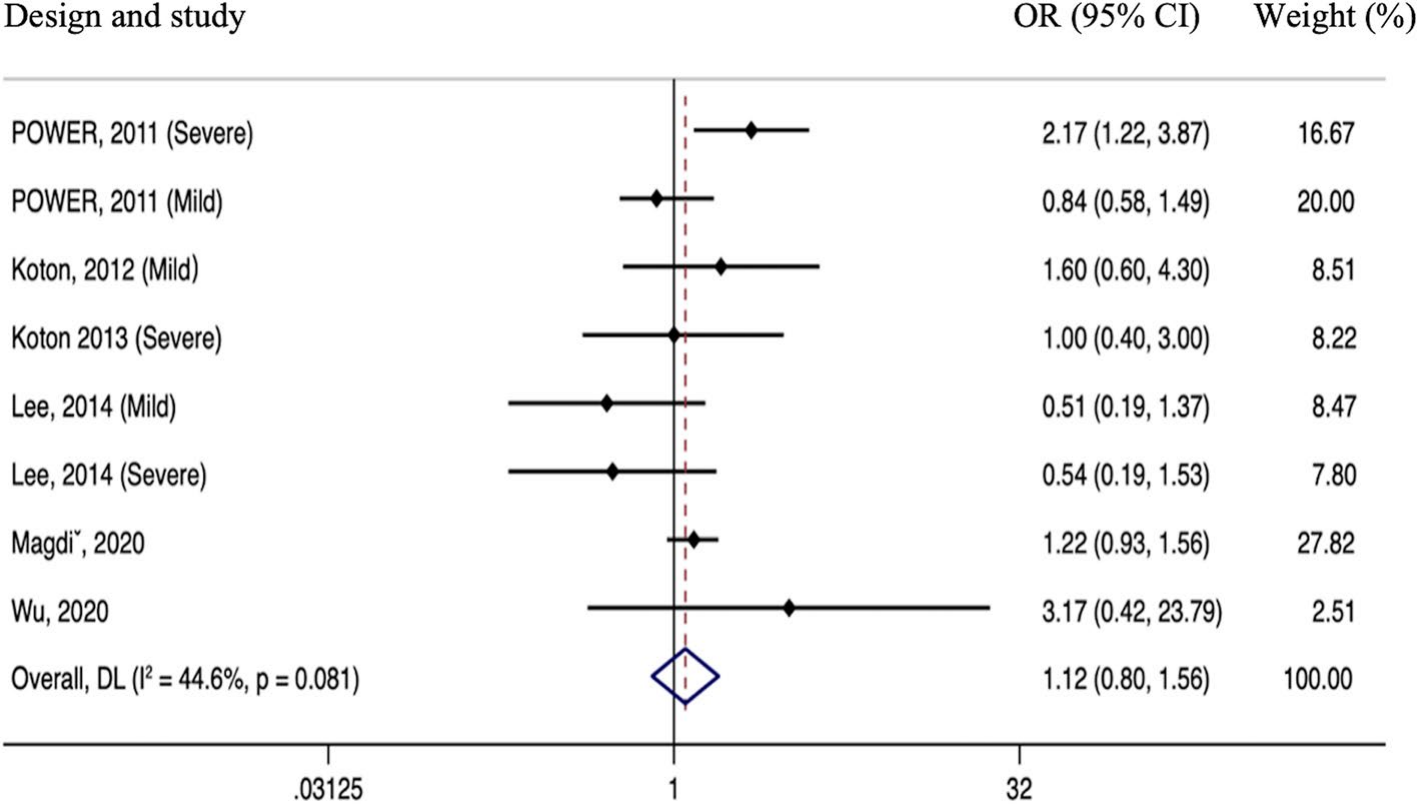
Meta-analyses of hazard ratios for the association between intracranial arterial calcification and stroke mortality

Five studies reported post-stroke mortality rates. Pooled results of the meta-analysis showed a lack of correlation between IAC and post-stroke mortality (pooled OR 1.12; 95% CI 0.80–1.56, *P* = 0.504) without significant heterogeneity among the studies (*P* = 0.081, I^2^ = 44.6%; Fig. 4).

### The results of subgroup analysis between IAC and ischemic stroke

In the study design subtype, a statistically significant effect of IAC on first stroke risk was observed in both cohort studies (OR 1.42; 95% CI 1.09–1.85, *p* <  0.001) and case-control studies (OR 1.79; 95% CI 1.12–2.863, *p* <  0.001). When stratified by continent, IAC was positively related with increased risk among studies performed in European (OR, 1.28; 95% CI 0.93–1.64, *p* <  0.001), North American (OR, 2.09; 95%CI 0.80–3.69, *p* = 0.001) and Asian (OR, 2.02; 95% CI 1.41–2.63, *p* <  0.001). In the further analysis by the number of cases, studies that included a small sample, that is, < 500 patients (OR 2.08; 95% CI 1.60–2.70, *p* <  0.01) had a higher risk of ischemic stroke than those studies with more than 500 cases (OR 1.33; 95% CI 0.99–1.79, *p* = 0.06). The results of subgroup analyses are shown in table 2.

**Table 2:** Summary risk estimates of the subgroup analysis results of ICAC and stroke occurrence

Subgroup	Studies (n)	OR (95%CI)	I^2^ (%)	*P* value
**Total**	8	1.62 (1.18, 2.23)	82.3	< 0.01
**Design**				
Case-control study	5	1.79 (1.12, 2.86)	88.4	< 0.01
Cohort study	3	1.42 (1.09, 1.85)	0	< 0.01
**Location**				
Asia	3	1.66 (0.90, 3.08)	91.3	0.10
Europe	3	1.52 (1.20, 1.92)	0	< 0.01
North America	2	2.09 (1.18, 3.69)	0	0.01
**Risk of bias**				
Low	3	1.41 (0.89, 2.25)	90	0.14
Moderate	5	1.66 (0.90, 3.08)	2.8	< 0.01
**No. of cases**				
< 500	4	2.08 (1.60, 2.70)	0	< 0.01
> 500	4	1.33 (0.99, 1.79)	73.9	0.06

Abbreviations: *OR* odds ratio, *CI* confidence intervals

### Publication bias

Visual inspection and Egger’s test indicated an asymmetric funnel plot (*p* = 0.007). As only eight studies assessing the risk of stroke occurrence were included in the present meta-analysis, we could not entirely rule out the presence of publication bias.

## Discussion

In this meta-analysis of studies on IAC and ischemic stroke, a positive correlation between IAC and stroke occurrence was found in the analysis (OR 1.62; 95% CI 1.18–2.23) and between IAC and stroke recurrence was also found (OR 1.77; 95% CI, 1.25–2.51). However, there was no significant correlation between IAC and post-stroke mortality.

IAC is a common incidental finding on brain CT scan in the general population and, although most of calcifications are viewed as innocent without any prognostic significance, others are related to adverse clinical outcomes [19–21]. Several studies showed that IAC was linked with an increased risk of stroke independently of risk factors for heart disease and other cardiovascular disease [22–24]. Although many diverse hypotheses have been proposed in previous studies, the mechanisms related IAC to ischemic stroke have not, as yet, been fully understood. First, vulnerable/unstable plaque with a large lipid-rich necrotic core (LRNC), thinning of the fibrous cap (FC), inflammation, and intraplaque hemorrhage (IPH), is more likely to rupture and may cause a brain ischemic event [25, 26]. Second, highly calcified arteries may, in some cases, have a severe narrowing or occlusion of the lumen leading to hemodynamic disturbances; also, the presence of large volumes of calcification reflects large plaques, which may be an essential source of embolic to the brain [27–29]. IAC may result in impaired arterial endothelial function, leading to damaged cerebral blood flow autoregulation and cerebrovascular reactivity, which might be a potential mechanism accounting for the lacunes and stroke [30–32]. In the current subgroup analysis stratified by region, IAC was positively related with increased risk among studies performed in European, North American and Asian, suggesting that IAC may be one of the most important risk factors for ischemic stroke worldwide. When stratified by study sample size, studies with small sample sizes seem to produce larger effect sizes than large studies.

Although this study showed that IAC was closely related to the recurrence of ischemic stroke, it must be note that there are still few studies in this fields and the conclusions are inconsistent. In the term of IAC impact on recurrence after ischemic stroke, Wu et al. showed a higher degree of IAC was associated with a high risk of stroke recurrence in patients with cerebral small-vessel disease, which might indicate chronic calcification observed in large intracranial arteries may have potential impacts on the cerebral vascular bed extending to small blood vessels [6]. Moreover, the predictive value for IAC for poor outcomes has not been established. Magdiˇ et al. reported that the presence of vertebrobasilar artery calcification was significantly related with the overall risk of long-term death and other cardiovascular events after ischemic stroke [14]. However, Koton et al. found that IAC was not significantly associated with mortality and poor functional outcome in patients with acute ischemic stroke after adjusting for traditional risk factors [16].

In our meta-analysis, no significant correlation was found between IAC and post-stroke mortality, still, the investigation of the link between IAC and stroke prognosis may have important clinical significance [33, 34]. Although there is still controversy regarding the relationship between calcification and instability of plaque, the amount and extent of multiple intracranial arterial calcifications can actually reflect the degree of evolution of atherosclerosis and the possibility of the presence of unstable plaques [35–37]. Furthermore, previous study showed that subjects with grade 3 or 4 calcification of an intracranial artery on brain CT were more likely to have a significant stenosis (greater than 50%) on cerebral angiography [38, 39]. At this point, IAC would be a useful marker for future cerebrovascular events after ischemic stroke, especially in non-cardioembolic infarction. Further evaluation of the potential causal link between calcifications in the intracranial vessels and poor outcomes after ischemic stroke is vital for future research to develop prevention strategies and implications.

To our best of our knowledge, this is the first systematic review and meta-analysis, focus on the relationships between IAC and ischemic stroke. As a common and easily identifiable finding on brain CT, IAC may be a useful indicator to predict the risk and outcome of ischemic stroke. Several limitations of this study should be considered. First, the studies included in this meta-analysis were either cohort studies or case-control studies, thus, the causality between IAC and the risk of ischemic stroke remains unclear. Second, the number of articles included seems too small, therefore, a larger-sized, prospective study is warranted to further investigate the association between arterial calcification and stroke prognosis. Third, we excluded articles that were not published in English to ensure the quality of studies, but it might lead to publication bias. Fourth, it would be very beneficial if subgroup analysis could be conducted based on the arteries involved. Although some recent studies aimed to explore the relationship between different patterns of intracranial arterial calcification and atherosclerotic disease [40], the exact patterns of involvement in the intracranial arteries and stroke were not reported in the included studies. Finally, different patterns of IAC may lead to different clinical outcomes, but all studies included in this meta-analysis did not report the relationship between IAC patterns and ischemic stroke.

## Conclusions

In conclusion, the findings from this study indicate an association between IAC and increased stroke risk. Further carefully designed and well-conducted studies with prospective design are needed to conduct to identify our results.

## Data Availability

All data generated or analyzed during this study are included in this article. Further enquiries can be directed to the corresponding author.

## Abbreviations

ICAS: Intracranial atherosclerotic stenosis
IAC: Intracranial arterial calcification
CT: Computed tomography
PRISMA: Preferred Reporting Items for Systematic Reviews and Meta-Analyses
RR: Risk ratio
OR: Odds ratios
CI: Confidence intervals
NOS: Newcastle-Ottawa Scale
LRNC: Large lipid-rich necrotic core
FC: Fibrous cap
IPH: Intraplaque hemorrhage.
